# Redox-sensitive factors as targets of thiol compounds to hinder SARS-CoV-2 replication and inflammatory response

**DOI:** 10.1128/aac.00518-26

**Published:** 2026-07-29

**Authors:** Marta De Angelis, Gianni Gori Savellini, Elena Piselli, Gabriele Anichini, Giulia Radocchia, Sofia Masini, Francesca Monittola, Francesca Bartoccini, Michela Bruschi, Mauro Magnani, Anna Teresa Palamara, Maria Grazia Cusi, Rita Crinelli, Alessandra Fraternale, Lucia Nencioni

**Affiliations:** 1 Department of Public Health and Infectious Diseases, Laboratory Affiliated to Istituto Pasteur Italia-Fondazione Cenci Bolognetti, Sapienza University 119653, Rome, Italy; 2 Laboratory of Virology, Department of Molecular Medicine, Sapienza University 315098 , Rome, Lazio, Italy; 3 Department of Medical Biotechnologies, Policlinico Santa Maria delle Scotte, University of Siena 574285, Siena, Italy; 4 Department of Biomolecular Sciences, University of Urbino Carlo Bo 204212, Urbino, Italy; 5 Department of Infectious Diseases, Istituto Superiore Di Sanità 9289, Rome, Italy; Chinese Academy of Medical Sciences & Peking Union Medical College, Beijing, China

**Keywords:** innate immunity, antiviral agents, inflammation, thiol compounds, oxidative stress, SARS-CoV-2

## Abstract

SARS-CoV-2 has undergone rapid genetic evolution, leading to the emergence of new variants with distinct mutations impacting global public health. Upon infection, the virus triggers a robust inflammatory response characterized by the release of pro-inflammatory cytokines, which play a central role in lung injury. It also alters the host antioxidant response, causing oxidative stress that supports viral replication and cytokine overproduction. This study investigated key pathogenic effectors in Calu-3 and A549-ACE2/TMPRSS2 cells infected with SARS-CoV-2 variants, focusing on replication kinetics, cellular redox state, and inflammatory cytokine profile. A dramatic redox alteration in terms of glutathione (GSH) and Cysteine (Cys) was observed at 48 h p.i., along with a strong pro-inflammatory cytokine response , likely via activation of the JNK/AP-1 signaling pathway. To counteract these effects, two thiol molecules were tested: I-152, a monothiol conjugate of N-Acetyl-Cysteine (NAC) and β-mercaptoethylamine (MEA) and its dithiol derivative, I-152SdAc. Thiols restored GSH balance by enhancing the expression of Nrf2-mediated genes, such as glutamate-Cys ligase modifier subunit (GCLM), and counteracted AP-1-mediated pathway, resulting in a significant reduction of inflammation and viral replication. Antiviral and anti-inflammatory activities of thiols were confirmed in NHBE cells. These findings highlight that redox imbalance is a key pathogenetic event in SARS-CoV-2 infection. Notably, besides Nrf2 and AP-1, other redox-sensitive factors, such as the CHAC glutathione-specific gamma-glutamyl-cyclotransferase 1 (CHAC1), seem to contribute to the pathogenesis and may represent a new potential therapeutic target of redox active compounds. Therefore, the thiol-derived molecules act as broadly effective compounds by limiting virus replication and inflammation.

## INTRODUCTION

SARS-CoV-2 has demonstrated rapid genetic evolution, resulting in the emergence of new variants, characterized by distinct mutations along the viral genome, with potential impact on global public health. As of early 2026, SARS-CoV-2 genetic diversity is characterized by the co-circulation of Omicron-derived lineages, primarily descendants of JN.1 (including KP sublineages), together with recombinant variants, such as XEC and XFG, all phylogenetically linked with the Omicron clade (1). The EG.5.1 variant is closely related to the XBB and contains a specific mutation known to evade some of the immunity gained from infection or vaccination (2–4). In this study, we selected EG.5.1, JN.1, and KP.3.1 as representative variants to investigate their distinct effects on viral infectivity and host-pathogen interactions. SARS-CoV-2 infection can trigger a cascade of events leading to an acute increase in circulating levels of different pro-inflammatory cytokines, including Interleukin-6 (IL-6), Interleukin-1β (IL-1β), and Tumor Necrosis Factor-α (TNF-α), which, if dysregulated, lead to lung damage, such as Acute Respiratory Distress Syndrome (ARDS) (5–10). Interestingly, it has been demonstrated that SARS-CoV-2 disrupts the intracellular redox homeostasis, contributing to lung damage (11–14). The nuclear factor erythroid 2-Related Factor 2 (Nrf2) is a crucial transcription factor in the redox-sensitive signaling pathway that helps protect cells from oxidative stress and damage (15). Some viruses target the host antioxidant response inhibiting Nrf2 in a replication cycle-dependent manner, supporting viral replication and increasing the inflammatory response (16–22). The inhibitory effect of SARS-CoV-2 infection on Nrf2 and downstream gene, e.g., Glutamate–cysteine ligase (GCL), Glucose-6-phosphate dehydrogenase (G6PD), and Heme Oxygenase 1 (HO-1) expression, and its involvement in lung damage has been demonstrated (23). Moreover, distinct SARS-CoV-2 proteins were observed to be involved in dysregulating redox homeostasis by interfering with the Nrf2 pathway (14, 24, 25). Strictly correlated with Nrf2 gene expression alterations may be the modified levels and metabolism of reduced glutathione (GSH) in the host cell, resulting in a loss of intracellular redox homeostasis (26). Considering the key role of redox pathways in regulating viral replication and inflammation, several studies suggest that restoring the intracellular redox conditions could effectively inhibit virus replication and reduce inflammation (27–29). Drugs with functional thiol groups have been widely used for their antioxidant and anti-inflammatory properties. These compounds exert their efficacy by altering the reduced/oxidized status of the Cys-rich SARS-CoV-2 structural proteins, such as the Spike glycoprotein (30). In previous works, the GSH-replenishing and antiviral activities of low molecular weight (LMW) thiols have been shown (30, 31). The LMW thiols previously studied and also included in this investigation were the monothiol I-152, a conjugate of N-acetyl-Cysteine (NAC) and S-acetyl-Cysteamine (SMEA) and the S-deacetylated dithiol derivative of I-152 (I-152SdAc) (32). The Calu-3 cell line served as a critical tool for studying various aspects of SARS-CoV-2 variants, including virus replication, pathogenesis, evolution, and drug development (33–35). Similarly, the engineered alveolar basal epithelial cells A549 ectopically expressing ACE2 and TMPRSS2 (A549-ACE2/TMPRSS2) were used as an alternative human cell model for virological investigations. Despite numerous studies on the characterization of SARS-CoV-2 infection with different variants, there is no direct comparison among viral variants covering virological, cellular redox impact, and innate immune response profiles. Our study aimed to analyze essential pathogenicity factors in Calu-3 and A549-ACE2/TMPRSS2 cells, focusing on replication kinetics, innate immune responses, cellular redox state, and the induction of pro-inflammatory cytokines. The redox alterations observed following the infection were not always correlated with an evident Nrf2 dysregulation but more likely with the upregulation of virus-induced factors involved in GSH degradation, i.e., glutathione-specific gamma-glutamyl-cyclotransferase 1 (CHAC1). I-152 and I-152SdAc induced Nrf2-dependent gene expression, i.e., γ-glutamate-Cysteine ligase (γ-GCL) that is rate-limiting in *de novo* GSH synthesis and downregulated CHAC1 expression. By these mechanisms, the thiol compounds restored a GSH-dependent redox balance, resulting in significant antiviral activity and anti-inflammatory properties, further confirmed in primary NHBE cells. The demonstrated efficacy of thiol-derived molecules in controlling inflammation and inhibiting viral replication highlights their potential for developing therapeutic strategies aimed at counteracting or ameliorating the ARDS caused by the cytokine storm induced by SARS-CoV-2 infection.

## RESULTS

### Thiol compounds impact SARS-CoV-2 replication in Calu-3 and A549-ACE2/TMPRSS2 cells

**Fig 1:**
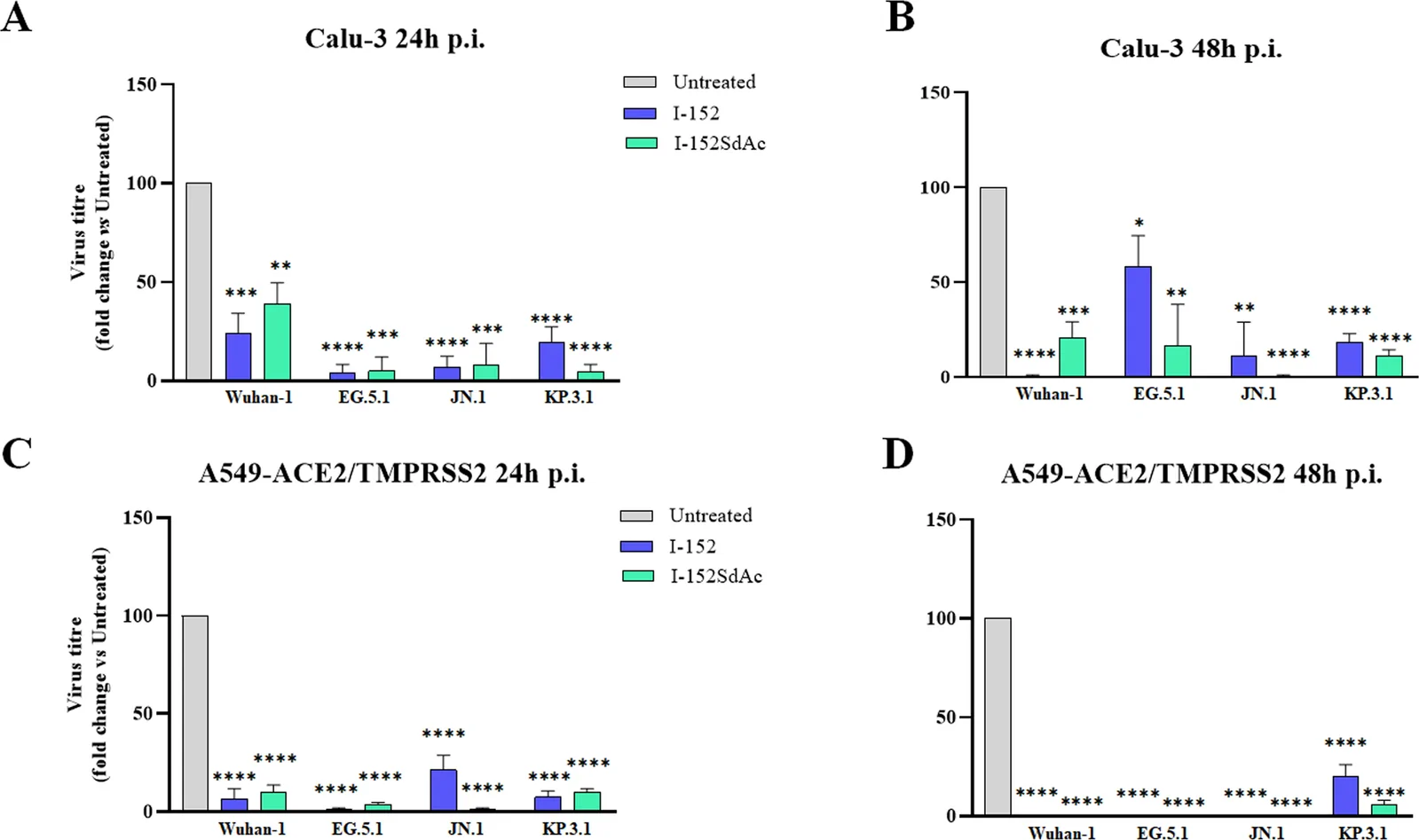
Inhibition of SARS-CoV-2 variants replication by thiol molecules. The antiviral activity of thiol compounds on Wuhan-1, EG.5.1, JN.1, and KP.3.1 SARS-CoV-2 strains was evaluated in Calu-3 cells at (**A**) 24 h and (**B**) 48 h post-infection (p.i.) in the presence of thiol-conditioned medium. Infected and untreated samples were included for each virus variant. Parallel experiments were conducted in A549-ACE2/TMPRSS2 cells under similar conditions at (**C**) 24 h and (**D**) 48 h p.i. Results are presented as the mean percentage of virus release ± standard deviation (SD) from at least three independent replicates (*n* ≥ 3). Statistical analysis was conducted using one-way ANOVA test with Tukey correction for multiple comparison, with significance thresholds indicated as *P* < 0.0001 (****), *P* < 0.01 (**), and *P* < 0.05 (*).

**Fig 2:**
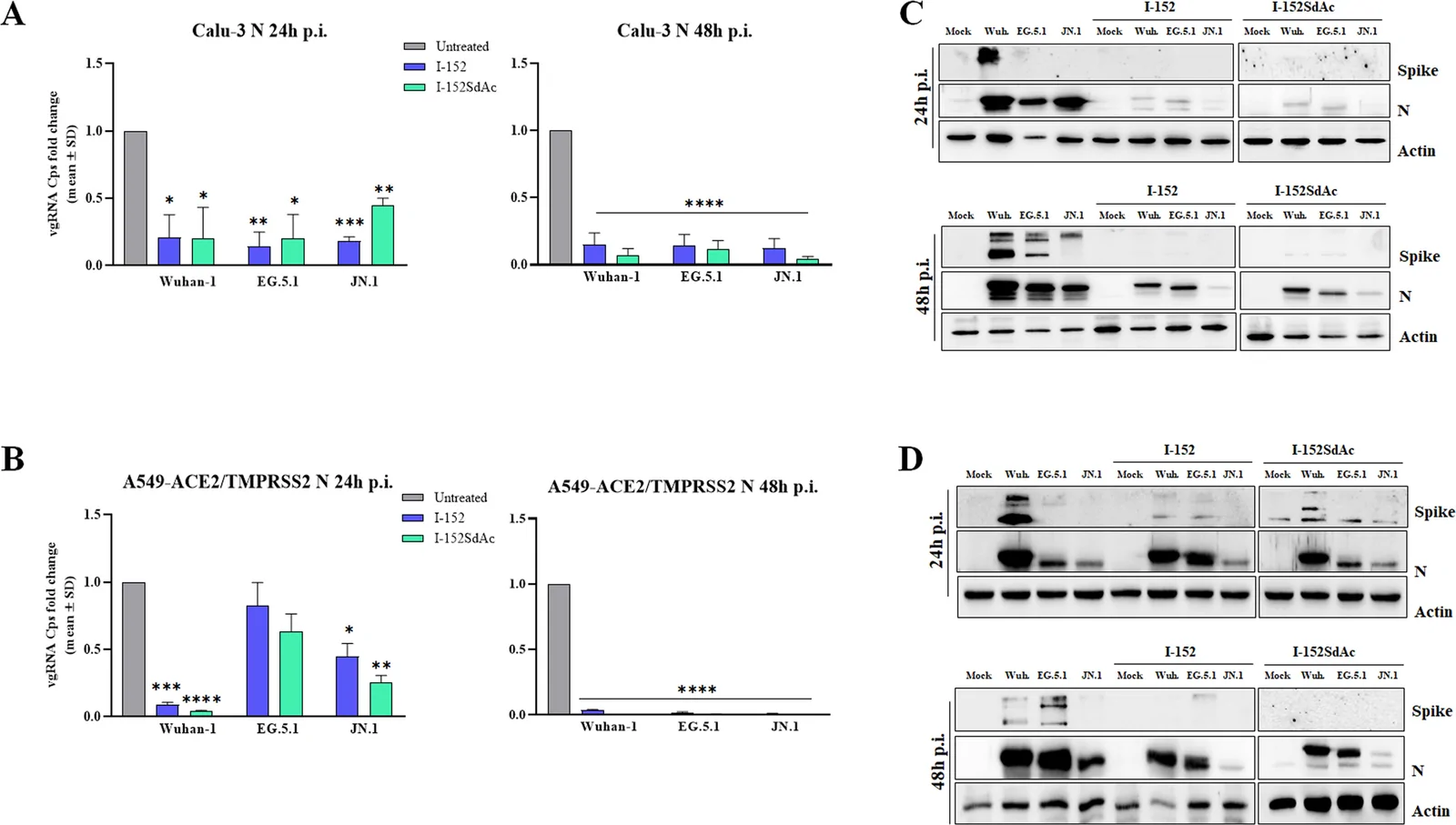
Inhibition of SARS-CoV-2 variants viral genome (vgRNA) copies production and protein expression by thiol molecules. Intracellular viral genome (vgRNA) copies (Cps) were quantified using RT-qPCR targeting the viral N gene in (**A**) Calu-3 and (**B**) A549-ACE2/TMPRSS2 cells collected at both 24 h and 48 h p.i. cultured in the presence or absence of thiol-conditioned medium. Results are presented as the mean fold change with respect to the untreated ones ± SD from at least three (*n* ≥ 3) independent replicates. Statistical analysis was conducted using a one-way ANOVA test with Tukey correction for multiple comparison, with significance thresholds indicated as *P* < 0.0001 (****), *P* < 0.001 (***), *P* < 0.01 (**), and *P* < 0.05 (*). Western blotting of Spike (S) and Nucleoprotein (N) viral proteins in whole cell lysates (30 µg) of infected (**C**) Calu-3 and (**D**) A549-ACE2/TMPRSS2 cells at 24 h and 48 h p.i., either in the presence or absence of thiol compounds. Images are representative of two (*n* = 2) independent technical replicates.

It has been previously observed that reducing agents significantly impair the infectivity and intracellular replication of SARS-CoV-2 Wuhan-1 strain in Vero E6 cells (30). Based on these observations, we explored whether I-152 and I-152SdAc also inhibit intracellular SARS-CoV-2 replication steps in human lung epithelial cells. Additionally, the antiviral activity of these compounds was tested on virus variants, such as EG.5.1, JN.1, and KP.3.1. To this aim, Calu-3 and A549-ACE2/TMPRSS2 cells were infected with different SARS-CoV-2 lineages, and the thiol molecules were added to the culture medium at a final concentration of 0.25 mM (30). Based on preliminary experiments (Fig. S1A and B), at low MOI (Multiplicity Of Infection) such as 0.01, the EG.5.1 and JN.1 virus lineages exhibited a reduced replication rate compared to the Wuhan-1 strain, in the selected cell systems and at the indicated times post infection (p.i.), leading to a misinterpretation of results, especially those regarding thiols’ antiviral activity. Therefore, a five-times higher MOI for EG.5.1 and JN.1 lineages, which resulted in an appreciable virus growth over time (Fig. S1C and D), was used for all the following experiments. Both I-152 and I-152SdAc significantly impaired viral replication at 24 h p.i. in Calu-3 leading to a reduction in viable virus release of 76% (*P* = 0.0008) and 61% (*P* = 0.017), respectively, for the Wuhan-1 strain and over 80% (*P* ≤ 0.0001) for the EG.5.1, JN.1, and KP.3.1 lineages (Fig. 1A). At 48 h p.i., a significant reduction in virus replication and release was still observed with both compounds (Fig. 1B). Similarly, the reducing agents significantly compromised SARS-CoV-2 variants replication in the A549-ACE2/TMPRSS2 cell system (Fig. 1C and D) at both time points. Although all the virus lineages replicated to high titers at 48 h p.i., thiols still exerted an antiviral activity at this time point (Fig. 1D). The antiviral effects of thiols were further confirmed in both cell systems by quantifying intracellular viral genome (vgRNA) copies of the Nucleocapsid (N) protein. As shown in Fig. 2, at both 24 h and 48 h p.i., the content of vgRNAs was significantly inhibited by both I-152 and I-152SdAc. However, the effects of thiol compounds on virus replication were more pronounced at 48 h p.i., in agreement with above results on viable virus release (Fig. 1). The beneficial properties of thiol molecules were confirmed by decreasing both intracellular viral genome copies and viable virus release. The inhibitory effects of I-152 and I-152SdAc on virus replication were further confirmed through western blot analysis of the viral Spike (S) and Nucleocapsid (N) proteins in Calu-3 cells infected with EG.5.1, JN.1, and Wuhan-1 strains showing higher viral fitness in terms of viral replication (Fig. 2C). Viral protein analysis revealed that the Spike protein of the EG.5.1 and JN.1 variants was undetectable at 24 h p.i., consistent with the limited virus replication observed in this cell system at this time point. Therefore, the inhibitory properties of the thiols could not be fully evaluated for the sub-lineages under the indicated experimental conditions (Fig. 2C; Fig. S2A). On the contrary, high levels of S protein were detected in the Wuhan-1 infected samples, which was considerably counteracted by thiols (Fig. 2C; Fig. S2A). As virus replication proceeded up to 48 h p.i., the S protein expression also became modestly detectable in EG.5.1 and JN.1 infected cell lysates (Fig. 2C; Fig. S2A). Nevertheless, the high inhibition of S protein expression mediated by I-152 and I-152SdAc was confirmed across all virus variants at 48 h p.i. (Fig. 2C; Fig. S2A). A different pattern was observed for the N protein, which was highly expressed at early time points (24 h p.i.) in all the infected samples, while treatment with both I-152 and I-152SdAc nearly abrogated its expression (Fig. 2C; Fig. S2A). This trend was maintained at 48 h p.i. although cellular expression and accumulation of the N protein significantly increased compared to the earlier time point (Fig. 2C; Fig. S2A). Similar findings were observed in A549-ACE2/TMPRSS2 cells (Fig. 2D; Fig. S2B). At 24 h p.i., the S protein expression was detectable and significantly diminished by thiol-derived molecules only in Wuhan-1-infected cells (Fig. 2D; Fig. S2B). Despite this, the S protein expression became barely detectable at 48 h p.i., and the antiviral activity of both I-152 and I-152SdAc was confirmed (Fig. 2D; Fig. S2B). The N protein was observed accumulating both at early and late times of infection, and its expression was negatively impacted by thiols in this cell system as well (Fig. 2D; Fig. S2B).

### Thiol compounds dampen SARS-CoV-2-induced cytokine response

**Fig 3:**
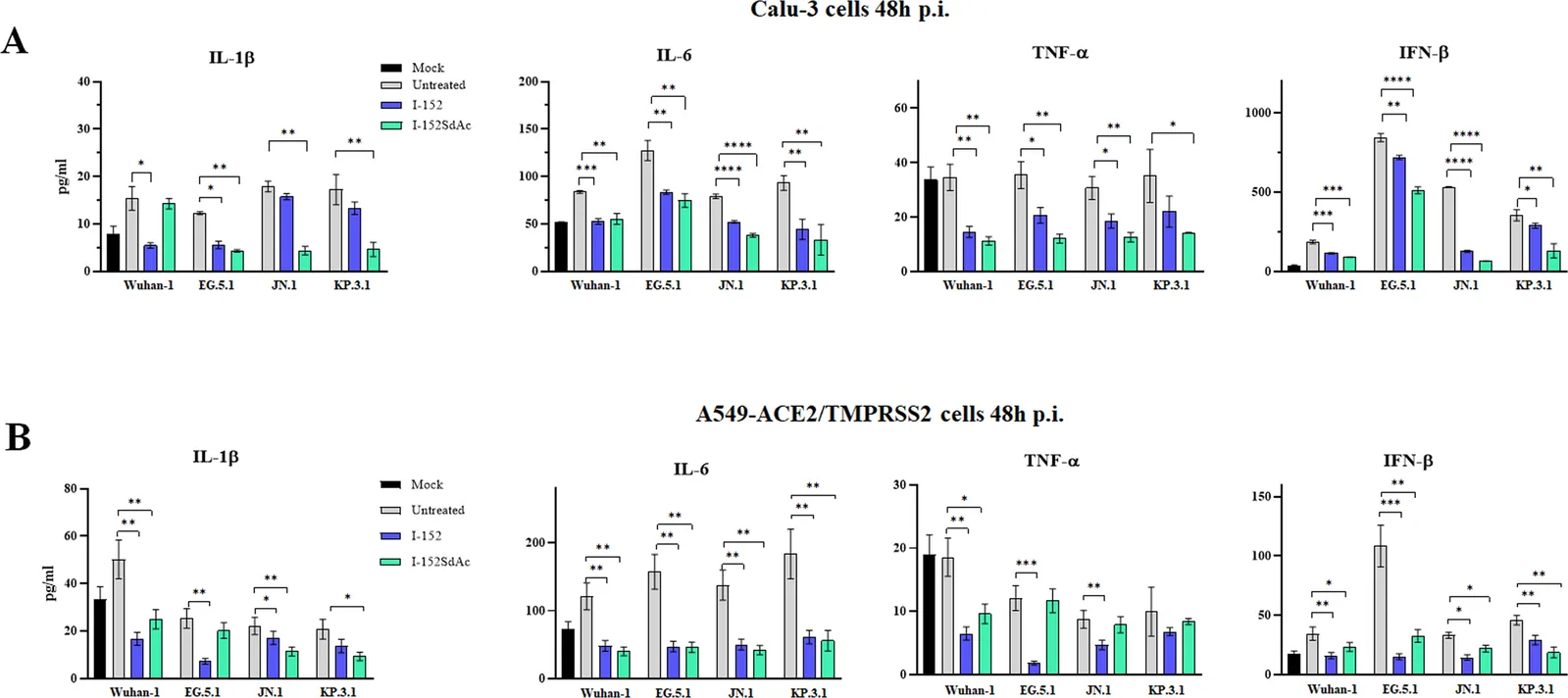
Suppression of inflammation induced by SARS-CoV-2 variants by thiol compounds. Interleukin-1 beta (IL-1β), Interleukin-6 (IL-6), Tumor Necrosis Factor alpha (TNF-α), and Interferon-beta (IFN-β) levels in the culture medium of (**A**) Calu-3 and (**B**) A549-ACE2/TMPRSS2 cells were quantified at 48 h p.i. using enzyme-linked immunosorbent assay (ELISA) based on standard curves. Data are presented as mean concentration (pg/mL) ± SD from at least three (*n* ≥ 3) independent experiments. Statistical analysis was conducted using a one-way ANOVA test with Dunn’s multiple comparison, with significance thresholds indicated as *P* < 0.0001 (****), *P* < 0.001 (***), *P* < 0.01 (**), and *P* < 0.05 (*).

First, we assessed the production of various immune mediators in Mock or infected, untreated or thiol-treated, Calu-3 cells, which is a more representative *in vitro* model for SARS-CoV-2 studies. Both the messenger RNA (mRNA) levels and the secreted amounts of the pro-inflammatory cytokines IL-1β, IL-6, IFN-β, and TNF-α were measured. The induction levels and timing of cytokine production differed depending on the SARS-CoV-2 variants. We observed a modest secretion of mature cytokines upon infection, in some cases, comparable to that of the Mock control at 24 h p.i. (data not shown). In contrast, a significant upregulation in the secretion of all the cytokines, except TNF-α, was observed at 48 h p.i. (Fig. 3A). This finding could be attributed to defects in the expression/induction of this cytokine in Calu-3 cells, in agreement with published data (35, 36). Moreover, processing of the mature, soluble form of TNF-α in response to stimuli is dependent on Tumor Necrosis Factor (TNF)-alpha converting enzyme (TACE) regardless of TNF-α transcription (37). Nevertheless, an anti-inflammatory activity of thiol compounds on basal TNF-α secretion was still observed, suggesting that I-152 and I-152SdAc retain beneficial properties even independently of the infection. As shown in Fig. S2A, the RT-qPCR assay revealed that all the tested virus lineages, at early times of infection (24 h p.i.), resulted in a significant increase in all cytokine expression at the transcriptional level except for IL-1β. At 48 h p.i., all the SARS-CoV-2 sub-lineages triggered considerable levels of the indicated mRNAs although each variant displayed distinct induction capabilities (Fig. S3B). Based on the *in vitro* antiviral potential of I-152 and I-152SdAc drugs against SARS-CoV-2, and on the capability of different virus lineages to stimulate inflammatory cytokines expression, we tested whether thiol drugs could be used to counteract this process. Both compounds reduced cytokine mRNA expression and protein secretion at both time points (Fig. S3A and B; Fig. 3A). Similar results were obtained in the A549-ACE2/TMPRSS2 cell line, highlighting the ability of all the tested viral strains to elicit a pronounced inflammatory response, at both transcriptional level and protein release, which is efficiently counteracted by thiol compounds (Fig. S3C and D; Fig. 3).

### Thiol compounds modulate SARS-CoV-2-induced inflammatory pathways

**Fig 4:**
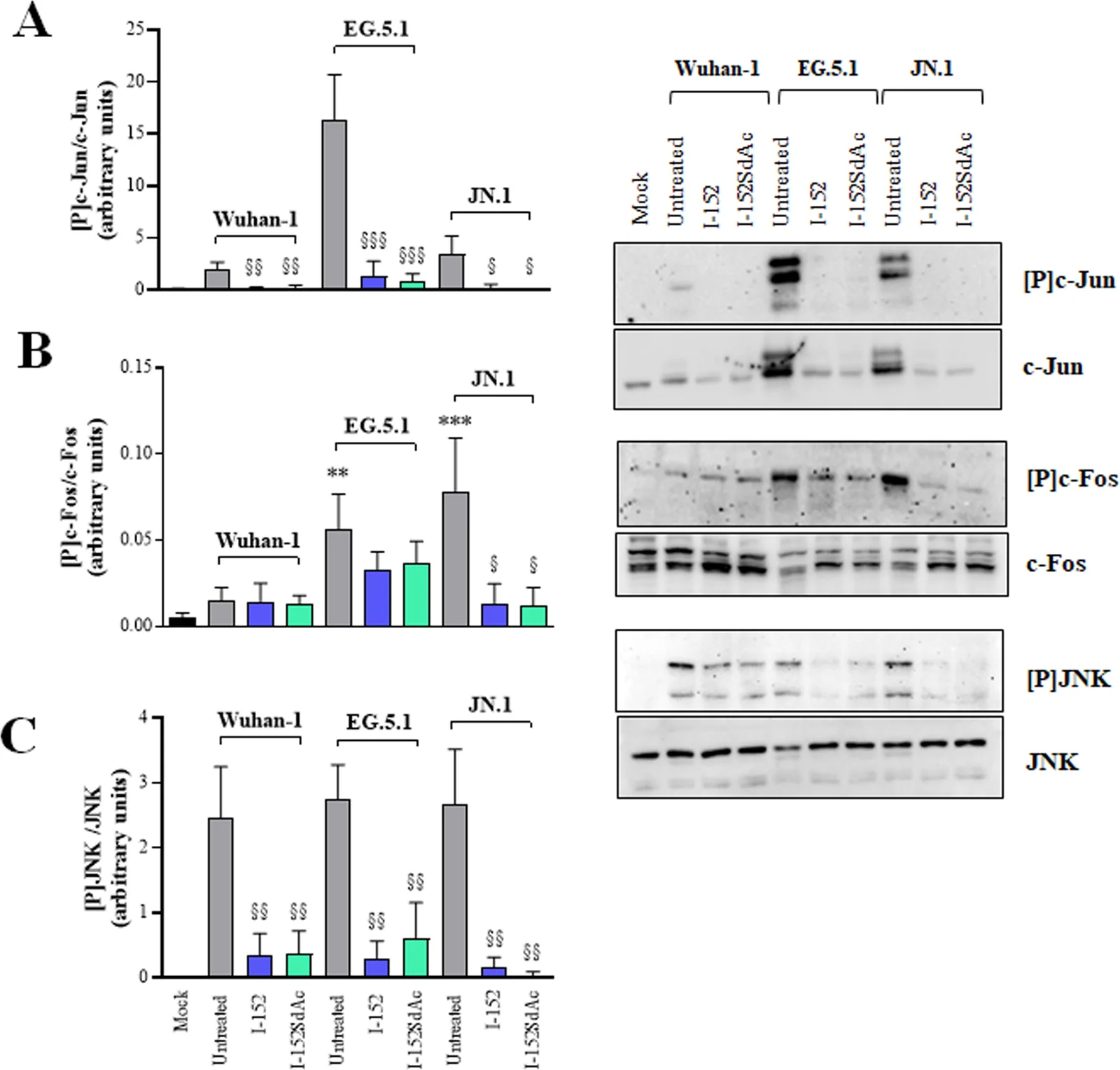
Regulation of AP-1 mediated inflammatory pathways in infected Calu-3 cells by thiol compounds. Whole cell lysates (10 μg) of Calu-3 cells, uninfected (Mock) or infected with SARS-CoV-2, untreated or thiols-treated, were loaded on SDS-polyacrylamide gels, and proteins were transferred to the PVDF membrane. After transfer, membranes were probed with (**A**) [P]c-Jun/c-Jun, (**B**) [P]c-Fos/c-Fos, and (**C**) [P]JNK/JNK antibodies. Phosphorylation level of each target was normalized to the relative total protein expression level. Results are reported in the graphs as the mean ± SD from three (*n* = 3) independent experiments. Statistical analysis was conducted using one-way ANOVA followed by the Dunnett’s multiple comparisons test with significance thresholds indicated as *P* < 0.001 (***/§§§), *P* < 0.01 (**/§§), and *P* < 0.05 (§); * significance *vs* Mock; § significance *vs* relative untreated cells.

Since the inflammatory response to the infection was effectively mitigated by the thiols, we investigated whether these molecules could exert direct effects on some basal cell signaling pathways that control inflammation. According to recent reports, SARS-CoV-2 and its individual proteins can activate not only the Nuclear Factor kappa-light-chain-enhancer of activated B cells (NF-κB) but also the Activator Protein-1 (AP-1) pathway to promote cytokine expression (38). For this reason, we analyzed phosphorylation status of p65 NF-kB subunit ([P]p65) which regulates its activity (39). Moreover, phosphorylated c-Fos ([P]c-Fos) and phosphorylated c-Jun ([P]c-Jun), which allow AP-1 to migrate into the nucleus and bind to its TPA Response Element (TRE) consensus regions, were evaluated. First, the activation of these pathways was investigated in Calu-3 cells infected with Whuan-1, EG.5.1, and JN.1 at 24 h p.i., albeit differences with respect to the Mock cells were not found (data not shown). At 48 h p.i., when peak production of the pro-inflammatory cytokines was observed, phosphorylation of NF-κB p65 was not markedly upregulated by the different virus strains, as demonstrated by comparable values of [P]p65/p65 ratio between the uninfected and infected samples (Fig. S4A). To dissect the mechanisms responsible for the inflammatory response triggered by the viruses, the levels of c-Jun and c-Fos, and the corresponding phosphorylated forms, were analyzed in the light of AP-1 incisive role in the inflammatory process during respiratory virus infections (40). Moreover, we have recently reported that thiol molecules can mitigate inflammation via the c-Jun/AP-1 and NF-κB pathways in LPS-stimulated macrophages (41). We found that the viruses, particularly the EG.5.1 and JN.1 strains, induced the phosphorylation of the AP-1 transcription factors c-Jun and c-Fos which was efficiently counteracted by the thiols, conceivably leading to reduced inflammation (Fig. 4A and B). Next, we examined the involvement of Jun N-terminal Kinase (JNK) since it is one of the major protein kinases responsible for increased expression of c-Jun and its phosphorylation (42). Western blot analysis of [P]-JNK revealed its activation in all the infected samples that, in turn, was effectively hampered by I-152 and I-152SdAc (Fig. 4C). Inflammatory pathways were also investigated in A549-ACE2/TMPRSS2 cells focusing only on one virus variant, e.g., the EG.5.1, as no lineage-specific differences were observed in the Calu-3 cell model. Herein, AP-1 activation was not as evident as in Calu-3 cells although [P]-JNK was markedly upregulated (Fig. S4B). Furthermore, the effect of I-152 on JNK phosphorylation was also confirmed (Fig. S4B). On the contrary, neither NF-kB nor c-Fos activation occurred in this cell line (Fig. S4B). This aspect could be related to a different AP-1 heterodimer composition among different cell lines, including homodimerization of c-Fos monomers, or the involvement of other Jun variants (JunB/JunD) or Musculoaponeurotic fibrosarcoma (MAF) family members (43). Taken together, these results suggest that the AP-1 transcription factor, rather than NF-kB, is the master regulator of inflammation during SARS-CoV-2 infections. In particular, c-Jun and c-Fos activation mainly occurs during SARS-CoV-2 variants infection and represents an important target of the thiol compounds. To support the role of AP-1 in the inflammatory process observed in our experimental conditions, we investigated Monocyte Chemoattractant Protein-1 (MCP-1) mRNA level in Calu-3 cells since its expression is known to be mainly regulated by MAPKs/AP-1 axis (44). We observed that MCP-1 was upregulated at 48 h p.i. and that the thiol molecules exerted its transcriptional repression, as already observed for the other cytokines (Fig. S4C). Finally, given the function of IFN-α/β in inducing the expression of IFN-stimulated genes via the Signal Transduction and Activator of Transcription 1 (STAT1) signaling pathway, we investigated STAT1 activation by measuring its phosphorylated levels ([P]STAT1) at 24 h p.i. (Fig. S4D) when IFN-β mRNA levels were higher with respect to all the other cytokines (Fig. S3A). As expected, the high IFN-β levels previously described led to STAT1 phosphorylation that, on the other hand, was massively reduced by the thiol molecules according to the reduced levels of IFN-β (Fig. S3A). Thus, the correlation between IFN-β expression and [P]STAT1 level was confirmed to occur during SARS-CoV-2 variants infection.

### Thiol molecules increase the Nrf2 expression in SARS-CoV-2 infected cells

**Fig 5:**
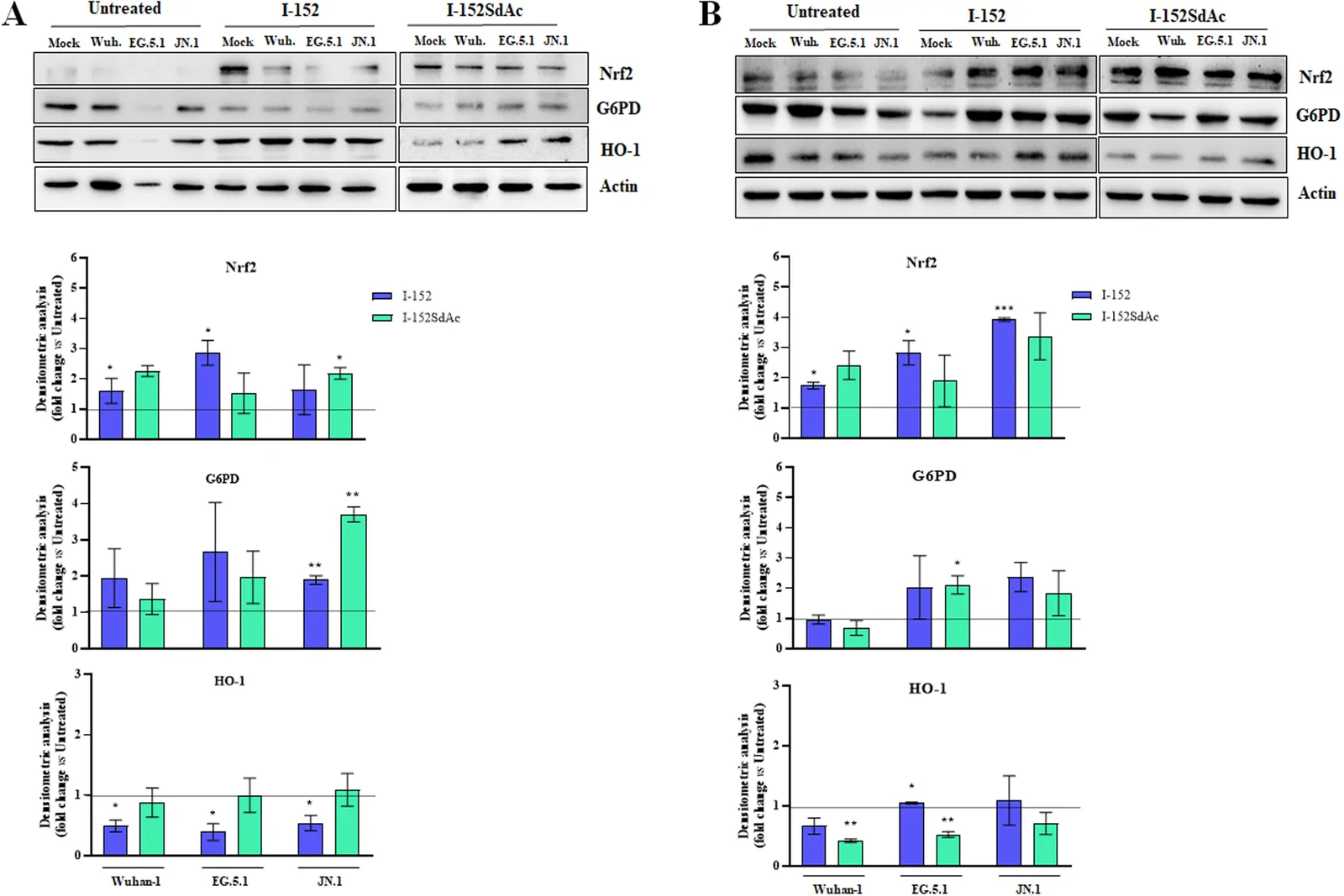
Regulation of Nrf2 and downstream scavengers by thiol compounds. (**A**) Nrf2 protein expression and its downstream antioxidant effectors were evaluated at 24 h p.i. in (**A**) Calu-3 and (**B**) A549-ACE2/TMPRSS2 cells either Mock-infected or infected with SARS-CoV-2 Wuhan-1, EG.5.1, or JN.1 lineages. Thirty micrograms of total proteins was separated on SDS-polyacrylamide gels and analyzed by Western blotting with antibodies against Nrf2, Heme Oxygenase-1 (HO-1), and Glucose-6-Phosphate Dehydrogenase (G6PD). Actin was used as a loading control, consistent with what is reported in Fig. 2, as the same WCLs were used for the detection of different target proteins. Samples treated with thiol-conditioned medium were analyzed in parallel. Images are representative of two (*n* = 2) technical replicates. Bar graphs represent densitometric analysis expressed as fold change of infected treated vs infected untreated conditions for each viral strain. Dashed lines (arbitrary value 1) represent infected, untreated, samples. Statistical analysis was conducted using a one-way ANOVA test with Dunn’s multiple comparison, with significance thresholds indicated as *P* < 0.001 (***), *P* < 0.01 (**), and *P* < 0.05 (*).

Nrf2 has a key role in protecting against stress-induced cell death, and it has, thus, been suggested as the master regulator of tissue damage during infection (45). Moreover, Nrf2 has been described as an important regulator of the inflammatory response (46). A significant redox imbalance characterizes SARS-CoV-2 infection, and several pieces of evidence suggest that manipulating redox-dependent pathways could serve as a promising therapeutic strategy against SARS-CoV-2 (21, 47). Therefore, taking advantage of the ability of I-152 and I-152SdAc to activate Nrf2 (48), we investigated in the same WCLs described above (Fig. 2C and D) how Wuhan-1, EG.5.1, and JN.1 strains modulated Nrf2 expression and its downstream signaling (24 h p.i.), both in the presence and absence of the thiol compounds. Dramatic changes in Nrf2 expression were not found in infected cells (Fig. 5). An overall increased Nrf2 and G6PD expression was observed with the treatments compared to the infected untreated Calu-3 cells (Fig. 5A). Interestingly, I-152 seems to exert an opposite effect on HO-1 expression, while I-152SdAc does not appear to influence HO-1 levels, possibly due to the involvement of alternative regulatory pathways independent of Nrf2 (49). Similar results were also observed in the A549-ACE2/TMPRSS2 cell line (Fig. 5B). The modulatory effects of the thiol-based compounds were also observed, even though to a lesser extent, at 48 h p.i. in Calu-3 cells (data not shown). These findings indicate that, although Nrf2 expression was not markedly altered by SARS-CoV-2 infection, treatment with thiol-containing molecules consistently enhanced Nrf2 expression and its downstream targets.

### Glutathione metabolism is impaired during SARS-CoV-2 infection, and thiol compounds restore the redox balance

**Fig 6:**
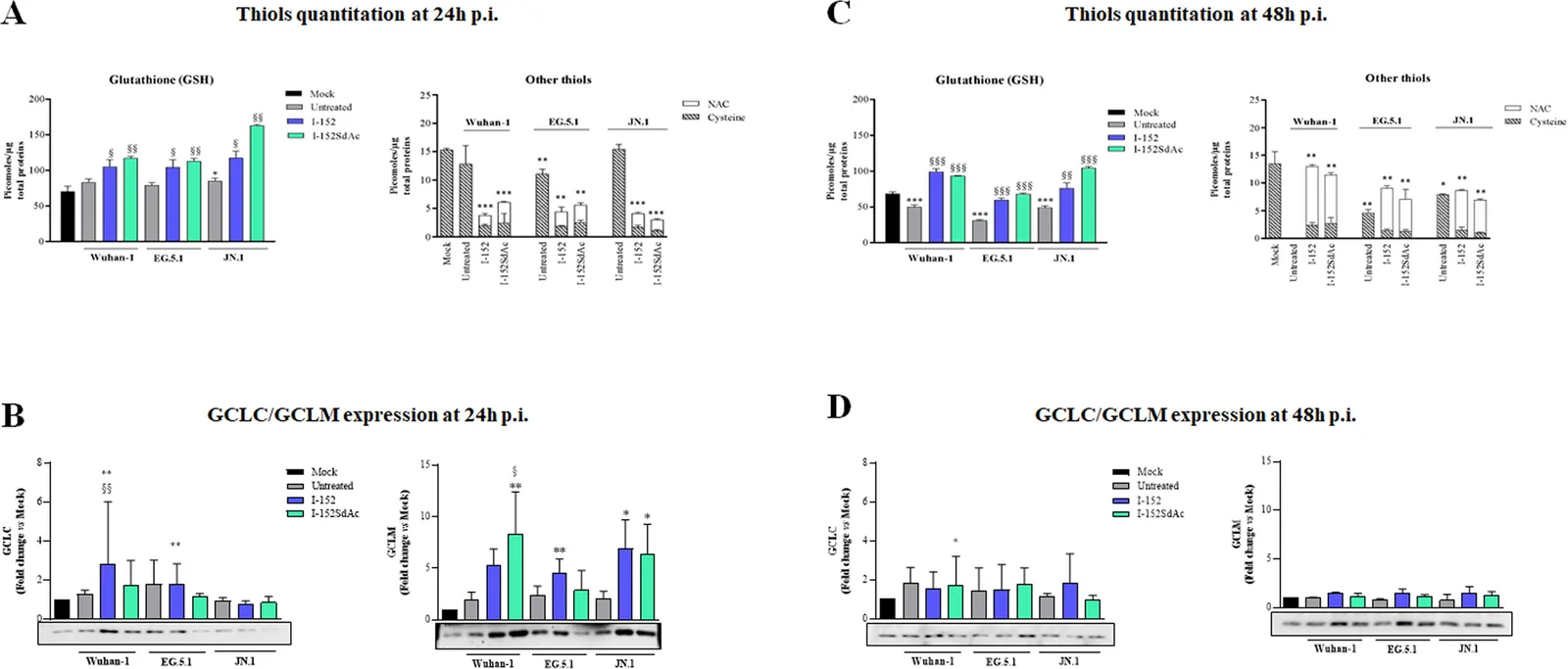
Modulation of glutathione (GSH) synthesis in infected Calu-3 cells either untreated or treated with the thiol molecules. Calu-3 cells were infected with SARS-CoV-2 Wuhan-1, EG.5.1, and JN.1 variants. Analyses were carried out at (**A and B**) 24 h and (**C and D**) 48 h p.i. GSH, as well as Cysteine and NAC, were quantified via high-performance liquid chromatography (HPLC) analysis, and the results are presented as the mean concentration expressed as picomoles/µg proteins ± SD of three (*n* = 3) independent experiments. Statistical analysis was performed with a Welch *t*-test. GCLC and GCLM protein expression (**B–D**) was analyzed by Western blotting. Cell lysates (5 μg) were separated on SDS-polyacrylamide gels, and proteins were transferred to PVDF membranes. Values were normalized to the total protein content as determined by the No-Stain Protein Labeling Reagent after transfer. GCLC and GCLM levels were expressed as the fold change compared to Mock and reported in the graphs as the mean ± SD from three (*n* = 3) independent experiments. Statistical analysis was conducted using one-way ANOVA followed by the Dunnett’s multiple comparisons test with significance thresholds indicated as *P* < 0.001 (***/§§§), *P* < 0.01 (**/§§), and *P* < 0.05 (*/§). * Significance vs Mock; § significance vs the relative untreated cells.

**Fig 7:**
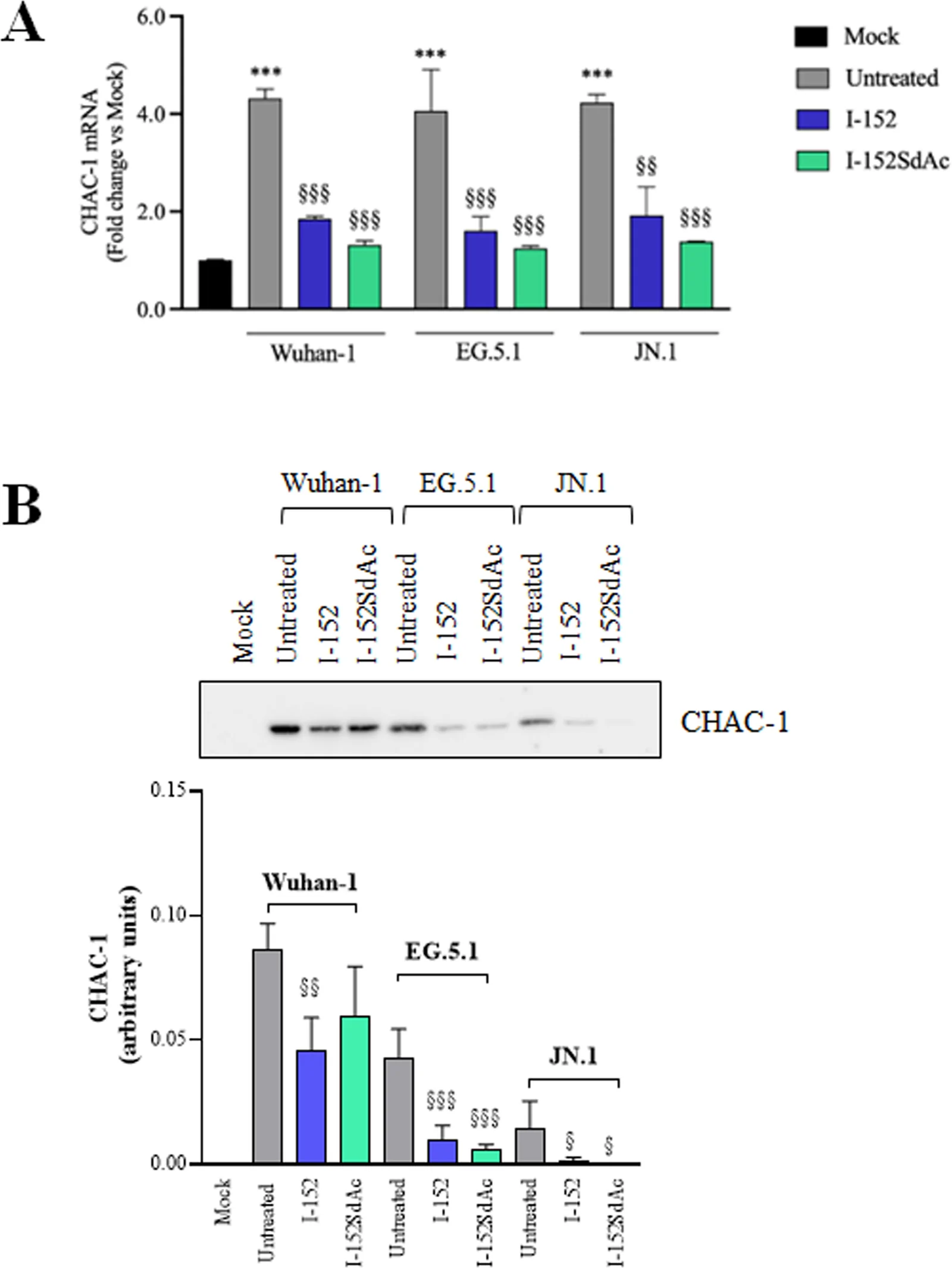
CHAC1 modulation by SARS-CoV-2 infection. Calu-3 cells, uninfected (Mock) or infected with SARS-CoV-2 Wuhan-1, EG.5.1, and JN.1 virus variants, were analyzed for CHAC1 (**A**) mRNA and (**B**) protein levels at 48 h p.i. Relative mRNA levels of CHAC1 were normalized against β2M and GAPDH and expressed as a fold change using the 2^−ΔΔCt^ method. For CHAC1 protein expression, cell lysates (20 µg) were separated on SDS-PAGE, and proteins were blotted with the specific antibody. Band intensity values were normalized to the total protein content as determined by the No-Stain Protein Labeling Reagent, and results were plotted in the bar graph as mean ± SD from three (*n* = 3) independent experiments. Statistical analysis was conducted using one-way ANOVA followed by the Dunnett’s multiple comparisons test with significance thresholds indicated as *P* < 0.001 (***/§§§), *P* < 0.01 (§§), and *P* < 0.05 (§). * Significance vs Mock; § Significance vs the relative untreated cells.

Impairment in the metabolism and redox function of cellular GSH during SARS-CoV-2 infection has been widely reported (26, 50). However, less information is available about the mechanisms by which SARS-CoV-2 induces GSH depletion and comparative studies between the ancestral Wuhan-1 strain and its variants in relation to their capacity to induce intracellular oxidative stress are absent. Among the markers that may be directly regulated by Nrf2, there are Glutamate-Cysteine Ligase (GCL) catalytic (GCLC) and modifier subunits (GCLM) of the heterodimeric enzyme eGCL that is the rate-limiting enzyme in the GSH synthesis (51). For these reasons, we analyzed GSH and Cysteine (Cys) levels as well as GCLC and GCLM expression. In Fig. 6, the results obtained by the evaluation of these parameters in Calu-3 cells infected with Wuhan-1, EG.5.1, and JN.1 strains, where the most significant redox changes were registered, are shown. At 24 h p.i., the redox perturbation induced by the viruses was efficiently counteracted by the maintenance of physiological GSH levels or even by its increase, e.g., during JN.1 infections (Fig. 6A). The major determinants of GSH synthesis are the availability of Cys. Therefore, Cys content was measured, and it was found decreased in all the infected samples, except in those with JN.1, compared to the Mock, suggesting that the amino acid may be depleted for *de novo* GSH synthesis (Fig. 6A). At the same time point, thiol treatments significantly increased GSH level in all the infected samples, induced Cys consumption, and provided N-Acetyl Cysteine (NAC) as possible Cys supplier (Fig. 6A). Different results were obtained 48 h p.i. (Fig. 6C). In fact, a dramatic decrease in GSH content was induced by all three virus variants. A marked reduction in Cys was also observed, becoming undetectable in Wuhan-1-infected cells. Overall, these findings suggest that a severe redox alteration occurs under all infected conditions that can no longer be compensated for by *de novo* GSH synthesis. On the contrary, the infected and treated samples had comparable or even higher GSH levels with respect to the Mock (Fig. 6C). Moreover, a high quantity of NAC deriving from the molecules’ metabolism was found. To dissect the mechanisms by which the virus strains and the thiol molecules can differently modulate the intracellular GSH content, GCLC and GCLM protein levels were studied. As shown in Fig. 6B and D, GCL subunits’ expression was not significantly modulated at any time p.i. The thiol compounds efficiently increased the expression of GCLM in infected samples at 24 h p.i. (Fig. 6B), while it reached almost basal levels at 48 h p.i. (Fig. 6D). GCLM upregulation has been reported to be particularly effective for GSH boosting (52). These results suggest that the molecules can effectively counteract the virus-induced oxidative stress by restoring/increasing GSH levels. However, these data do not explain the significant GSH depletion registered in the infected cells at 48 h p.i. Therefore, we turned our attention to ChaC glutathione-specific gamma-glutamyl-cyclotransferase 1 (CHAC1), which is an ER stress-inducible gene that is involved in the degradation of glutathione (53). We investigated CHAC1 expression in Calu-3 cells, and we found that its expression was not modulated at early times of infection (24 h p.i.) by all the virus lineages tested (data not shown). Conversely, CHAC1 was significantly upregulated at 48 h p.i. (Fig. 7), in parallel with the GSH depletion previously detected in all the tested samples (Fig. 6C). In addition, the presence of thiol molecules reduced the accumulation of CHAC1, thereby restoring a basal cellular state and preventing virus-induced cell damage. To confirm the role of CHAC1 in SARS-CoV-2-induced redox alterations, CHAC1 protein expression was studied in A549-ACE2/TMPRSS2 cells and in Calu-3 cells infected with the KP3.1 virus variant (Fig. S4E). Overall, these findings highlight a role for CHAC1 in modulating the host redox state during SARS-CoV-2 infection, suggesting that it may contribute to the cellular pathways involved in pathogen-induced GSH depletion and consequent oxidative stress induction and cellular dysfunction.

### Thiol molecules inhibit SARS-CoV-2 replication and pro-inflammatory cytokine production in human primary NHBE cells

**Fig 8:**
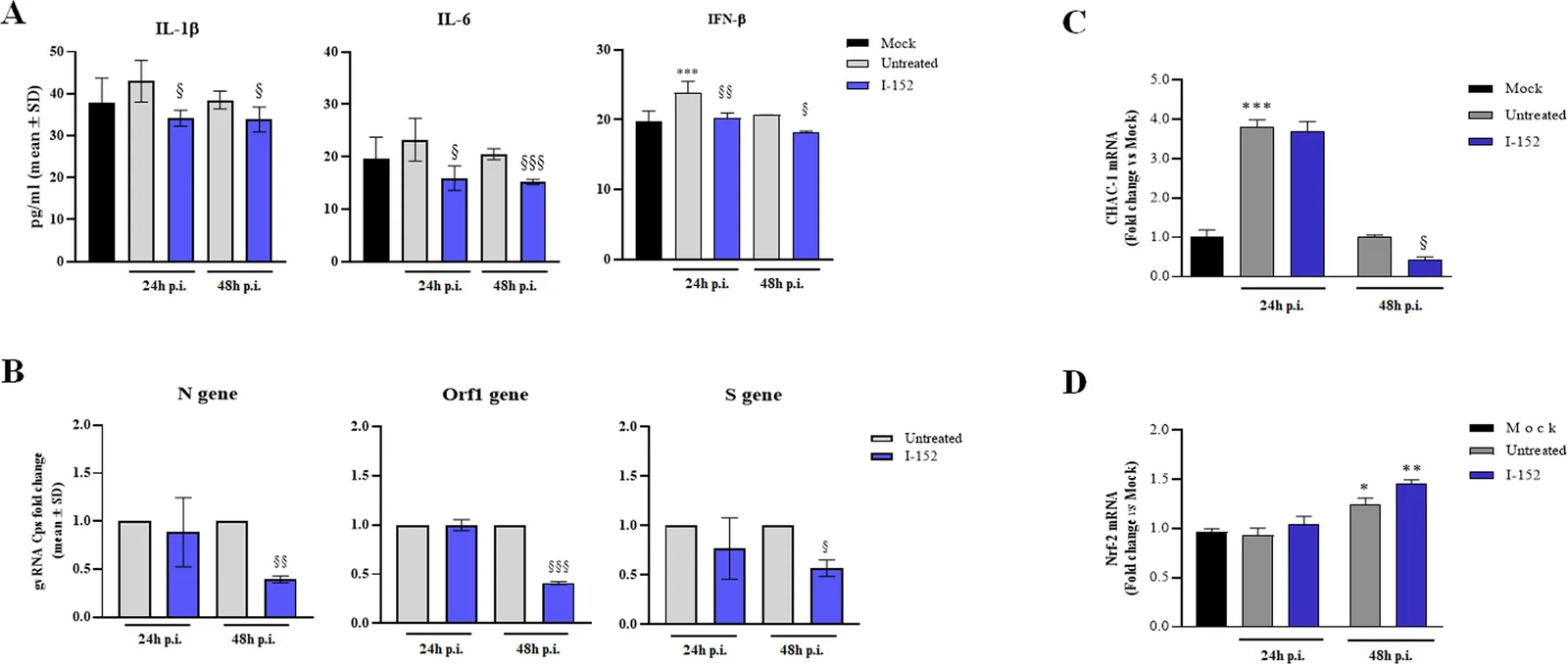
Antiviral and anti-inflammatory activity of I-152 in NHBE primary cells. Interleukin-1 beta (IL-1β), Interleukin-6 (IL-6), and Interferon-beta (IFN-β) levels (**A**) were quantified in the culture medium of uninfected (Mock) or Wuhan-1-infected Normal Human Bronchial Epithelial (NHBE) cells treated or not with I-152 and collected at 24 h and 48 h p.i. Data are presented as mean concentration (pg/mL) ± SD from two (*n* = 2) independent experiments. Intracellular viral genome (vgRNA) copies (Cps) (**B**) were quantified using RT-qPCR targeting the viral N, Orf1, and S genes in total RNA isolated from NHBE cells treated as described above. Results are presented as the mean fold change in vgRNAs of I-152-treated infected samples with respect to the untreated ones ± SD from two (*n* = 2) independent replicates. The same total RNA samples were used to assess relative CHAC1 mRNA levels (**C**) and Nrf2 (**D**) by RT-qPCR. Statistical analysis was conducted using a one-way ANOVA test with Dunn’s multiple comparison, with significance thresholds indicated as *P* < 0.001 (§§§), *P* < 0.01 (§§), and *P* < 0.05 (§). * Significance vs Mock; § Significance vs the relative untreated cells.

The antiviral and anti-inflammatory activities of thiol molecules were also partially investigated in Normal Human Bronchial Epithelial (NHBE) primary cells infected with Whuan-1. In this cell system, we used the experimental conditions previously applied to the other cell lines, including the virus infectivity dose (MOI = 0.01) and the molecule treatment conditions (0.25 mM I-152). The results demonstrated that Wuhan-1 infection led to a modest, not significant, effect on most of the investigated pro-inflammatory cytokines, with the exception of IFN-β (Fig. 8A). Notwithstanding, treatment with I-152 modulated the inflammatory response over time (Fig. 8A). However, we can speculate that the undetectable cytokine modulation could be a consequence of the modest virus replication, likely due to the low infectious dose used. Indeed, it was not possible to assess virus replication/inhibition by titration method. Therefore, we assessed molecular analysis for viral genome copy number determination. RT-qPCR further revealed that I-152 decreased the expression of viral N, Orf1, and S genes, particularly at 48 h p.i., suggesting the inhibition of viral replication (Fig. 8B). Additionally, as a marker of virus-induced cellular distress, CHAC1 and Nrf2 mRNAs expression was quantified. CHAC1 mRNA was found significantly elevated in untreated infected cells at 24 h p.i., whereas I-152 treatment reduced this expression and led to CHAC1 mRNA marked decrease by 48 h p.i (Fig. 8C). Conversely, Nrf2 expression was upregulated by virus infection and replication at 48 h p.i., an effect which was significantly enhanced by the I-152 molecule to restore cellular redox state (Fig. 8D). Together, these findings confirmed that even in primary cells thiol molecules exert antiviral and immunomodulatory effects by limiting virus replication and dampening excessive inflammatory responses during SARS-CoV-2 infection.

## DISCUSSION

Impairment of antioxidant systems, with a significant role of Nrf2, may contribute significantly to the pathogenesis of SARS-CoV-2 infection (14, 16, 21). Thiol-based drugs have been previously demonstrated to target viral components and host factor activity involved in virus protein maturation (28, 30, 54). Indeed, targeting cellular functions can also provide opportunities not only to hamper viral replication but also to moderate deleterious host responses and protect from tissue injury.

In the present work, we investigated the modulation of antioxidant and inflammatory response in human airway epithelial cells by SARS-CoV-2 Wuhan-1 strain and some Omicron representative variants. In parallel, the antiviral and anti-inflammatory activities of I-152 and its dithiol derivative I152SdAc were evaluated. Indeed, previous findings demonstrated that I-152 increases Nrf2 expression leading to transcriptional induction of downstream scavenger genes to limit the inflammatory response to Lipopolysaccharide (LPS) (41, 48). Consistently, in this work, we observed that I-152 and I-152SdAc inhibit both SARS-CoV-2 replication, as well as the expression of the main inflammatory cytokines. About the antiviral mechanism of the thiol compounds, we have already demonstrated in Vero E6 cells that they inhibit both SARS-CoV-2 (Wuhan-1 strain) virus entry and mature virion production by interfering with viral protein folding (30). As confirmed in this study, booster of infected bronchial (Calu-3) and alveolar (A549-ACE/TMPRSS2) cell lines or Normal Human Bronchial Epithelial (NHBE), which has gained prominence as an alternative to animal models (55–58), with thiols efficiently inhibited virus replication. Importantly, molecular analyses demonstrated that I-152 significantly reduced viral genome copy numbers (N, Orf1, and S viral genes), particularly at 48 h p.i., confirming its antiviral activity in primary airway cells. Together, these data confirm that disruption of redox equilibrium is a key factor in SARS-CoV-2 replication and host cell entry (21, 23, 59).

Furthermore, thiol compounds may be linked to the release of H_2_S from cysteines, which has a deleterious impact on SARS-CoV-2 infection, as recently reported (29, 60). Our results suggest that both the ancestral Wuhan-1 strain and the Omicron variants have the potential to interfere with pathways controlling the antioxidant and cytoprotective cellular machinery. Calu-3 cells infection leads to a dramatic GSH and Cys drop at 48 h p.i despite unaffected expression of Glutamate-Cysteine Ligase (GCL), that is the rate-limiting factor for the synthesis of the tripeptide (52), suggesting a mechanism for the dynamic regulation of GSH biosynthesis within the cell. Indeed, only in the early time of infection (24 h p.i.), the cellular antioxidant machinery can counteract the oxidative stress induced by infection maintaining intracellular GSH homeostasis. We can hypothesize that at early phases of the viral life cycle, the cell’s GSH biosynthetic apparatus can synthesize the tripeptide due to Cys availability (61). At 48 h p.i., when the GSH content was significantly decreased, the Cys level also dramatically dropped, limiting the potential of the host cell to support the *de novo* GSH biosynthesis (26, 62). In such a condition, an increase in Cys concentration is expected to boost GSH synthesis. NAC, slowly released by I-152, could represent an effective Cys supplement. Moreover, an induction of the Nrf2-dependent GCL modifier subunit (GCLM) expression was observed in the thiol-treated samples, and this could represent the major homeostatic mechanism for intracellular redox balance, as previously reported (46, 62, 63). Together, these data highlighted the mechanisms by which thiols can increase GSH: on one hand, they boost its rate-limiting substrate Cys (as revealed by high NAC concentrations); on the other hand, they augment GSH levels through increased Nrf2-dependent expression of its synthetic enzymes (GCLC/GCLM). The cellular metabolism of GSH is also strikingly dependent on CHAC glutathione-specific gamma-glutamyl-cyclotransferase 1 (CHAC1), which, therefore, has been identified as an essential regulator of oxidative stress (64). Our data showed, for the first time, that CHAC1 is upregulated both in Calu-3, A549-ACE2/TMPRSS2 cell lines and in primary NHBE by SARS-CoV-2 infection, potentially contributing to GSH depletion observed upon infection and assuming a central role in viral replication. Meanwhile, the treatments decreased CHAC1 expression further contributing to redox homeostasis restoration. Although the very preliminary role of CHAC1 in SARS-CoV-2 replication will need further characterization, such as by siRNA-mediated or CRISPR/Cas9 CHAC1 depletion, we can speculate that it may be considered a potential new therapeutic target for SARS-CoV-2 infection treatment. Along with CHAC1 involvement in the mechanisms through which SARS-CoV-2 alters host cell responses, our data highlight the central role of redox imbalance in COVID-19, suggesting that several redox-sensitive factors may be promising therapeutic targets to counteract both viral replication and cytokine storm. In fact, we demonstrated that the cytokine peak correlated with GSH depletion. Based on these observations and considering the efficacy of Nrf2 agonists on both viral replication and inflammatory markers (47), we also investigated how the thiol compounds could counteract the pro-inflammatory response. It is well known that SARS-CoV-2 or its single proteins induce aberrant NF-κB activation and AP-1-mediated mechanisms to induce a pro-inflammatory response in the pulmonary epithelial cells and immune cells (38, 65, 66). Our study supported the incisive role, in a virus lineage-independent manner, of AP-1 in modulating the inflammatory response of Calu-3 cells to the infection. In fact, the increased levels of c-Fos and c-Jun phosphorylation during virus infection, which is involved in AP-1 transcriptional activity (67), perfectly correlated with IL-6, IL-1β, TNF-α, and IFN-β inflammatory cytokine transcriptional expression and release (68). Furthermore, since AP-1 is a redox-sensitive transcription factor that modulates gene expression responses to oxidative stress, it represents an additional potential target for the I-152/I-152SdAc, which restricts the activity of upstream kinases (i.e., JNK/c-Jun) (69, 70). Further supporting evidence was provided by the consistent upregulation of Monocyte Chemoattractant Protein-1 (MCP-1) following SARS-CoV-2 infection and by its subsequent restoration to physiological levels upon thiol treatment. Notably, MCP-1 expression is predominantly regulated by AP-1 (71). In Calu-3 and A549-ACE2/TMPRSS2 cell lines, as well as primary NHBE cells thiols treatment upon SARS-CoV-2 infection was able to strongly counteract the inflammatory response, suggesting that they could represent valid tools to reduce the severity of SARS-CoV-2 infection and excessive inflammatory response. Moreover, this approach would overcome the problem linked to the continually evolving virus variants offering new means for developing therapeutics against both existing and emerging viral threats.

Despite experiments on NHBE being performed using cells derived from a single donor, limiting potential inter-individual variability, the generalization of the observed responses of the indicated cell system to the infection and to thiols' antiviral/anti-inflammatory properties was confirmed. In addition, the use of a very low viral inoculum resulted in undetectable levels of virus release over time. Therefore, viral replication and the antiviral activity of thiols were assessed by quantifying intracellular viral genome copy numbers. Although RT-qPCR is not considered the gold-standard method for viral infectivity assessment, several lines of evidence support a correlation between RT-qPCR quantification and viral titration assays (72, 73). To overcome inconclusive results and strengthen the assay, three different viral genes (N, S, and Orf1) were selected for quantification. Notwithstanding, our findings demonstrate that the antiviral and immunomodulatory properties of I-152 and I-152SdAc molecules are preserved in primary epithelial cells, reinforcing their potential relevance *in vivo*. Indeed, major challenges remain to be overcome in order to expand the clinical applications of these compounds, such as the need for careful safety and efficacy investigations in animal models.

In conclusion, these compounds could represent small broad-spectrum antivirals able to hamper a specific stage of the viral life cycle, i.e., protein folding, and to moderate deleterious host responses, which play key roles in the pathogenesis of several viral infections, in particular by respiratory viruses (74–76).

## MATERIALS AND METHODS

### Cells and viruses

Human Calu-3 lung epithelial cells (ATCC HTB-55) were cultured in Dulbecco’s modified Eagle’s medium (DMEM) (EuroClone, Milan, Italy) supplemented with 100 U/mL penicillin/streptomycin (EuroClone) and 10% heat-inactivated fetal bovine serum (FBS) (EuroClone) at 37°C. Vero E6 (ATCC CRL-1586) cells were maintained in DMEM supplemented with 5% FBS, while A549-ACE2/TMPRSS2 cells (The National Institute for Biological Standards and Control, NIBSC, #01003) were cultured in DMEM containing 10% FBS, supplemented with 2 mg/mL G418 and 200 μg/mL hygromycin. Normal Human Bronchial Epithelial (NHBE) cells (Lonza, Milan, Italy) were cultured in Bronchial Epithelial Growth Media (BEGM) BulletKit (Lonza) as suggested by the manufacturer. The SARS-CoV-2 lineages were isolated on Vero E6 cells from clinical specimens (according to the principles of the Declaration of Helsinki, with reference to BIOBANK-MIU-2010 document approved by the Ethics Committee with amendment No. 1, on 17 February 2020) at the Virology Unit of the Policlinico “S. Maria alle Scotte” in Siena, Italy. Prior to participating in this study, all subjects signed a written informed consent. Isolates of the Wuhan-1, EG.5.1, JN.1, and KP.3.1 lineages were sequenced using the COVIDSeq test (Illumina, Milan, Italy) following the manufacturer’s recommendations (GenBank accession numbers MT531537, PQ565585, and PQ565587, respectively).

### Reagents

Geneticin (G-418) and hygromycin were purchased from TCI, Zwijndrecht, Belgium. I-152 and I-152SdAc were synthesized as previously described (32). Both thiol molecules were used in all the experiments at a final concentration of 0.25 mM and were freshly prepared in complete DMEM containing 5% FBS and filtered for sterilization.

### SARS-CoV-2 infection

All the experiments with SARS-CoV-2 were performed in the Biological Safety Level 3 (BSL-3) laboratory of the Policlinico “S. Maria alle Scotte” Hospital in Siena, Italy. Preliminary viruses’ kinetic growth in Calu-3 or A549-ACE2/TMPRSS cell lines was conducted to set up the appropriate infectious dose for further experiments. Thereafter, the indicated cell lines were seeded at 2 × 10^5^ cells per well in a 24-well plate in complete culture medium. The day after, cultures were mock-infected or infected with a MOI of 0.01 of the Wuhan-1 or KP.3.1 SARS-CoV-2 strains or with a MOI of 0.05 of the EG.5.1 or JN.1 lineages for 1 h at 37°C. Accordingly, NHBE cells were infected with MOI of 0.01 of the Wuhan-1 strain. After extensive washing to remove the inoculum, DMEM containing 5% FBS or BEGM complete growth medium, supplemented or not with the indicated thiol molecules, was added. Supernatants were collected at 24 h and 48h post-infection (p.i.) and stored at −80°C for further analysis.

### Determination of residual viral load

To assess the antiviral activity of I-152 and I-152SdAc, supernatants of Calu-3, A549-ACE2/TMPRSS2, or NHBE cells described above were tested for residual viral particle by virus microtitration assay on Vero E6 cells in 96-well plates. Ten-fold dilutions from each sample were used to infect cell cultures in four replicates. The cytopathic effect (CPE) was observed under a light microscope 3 days p.i. The Reed-Muench formula was used to calculate viral titers as TCID_50_/mL. Viral genomic (vgRNA) intracellular copies (Cps) were determined by Reverse-Transcription quantitative PCR (RT-qPCR). Briefly, total RNA samples from infected cells collected at the indicated times p.i. were prepared using the RNeasy Plus Mini kit (Qiagen, Milan, Italy) and analyzed with the TaqPath COVID-19 RT-qPCR Kit, which includes primers/probe for N gene and, where indicated, for S and ORF1ab genes, along with glyceraldehyde-3-phosphate dehydrogenase (GAPDH) (Applied Biosystems, Milan, Italy). A 25 μL reaction was set up containing 100 ng of total RNA, following the manufacturer’s instructions. Each assay was performed in triplicate on the Applied Biosystems 7500 Real-Time PCR System (Applied Biosystems). Experimental vgRNA Cps were calculated using the 2^−ΔΔCt^ algorithm using GAPDH for normalization. Results were reported as mean fold reduction in vgRNA Cps ± standard deviation from at least three independent experiments, with mock-treated infected samples representing the reference.

### Assessment of oxidative stress markers and cytokine expression via RT-qPCR

Cell pellets from treatments described above were subjected to total cellular RNA purification using the RNeasy PLUS Mini Kit (Qiagen) following the manufacturer’s recommendations. The quality and quantity of the extracted RNA were verified by spectrophotometric reads, and equal amounts (100 ng) of RNA were subjected to Reverse-Transcription quantitative PCR (RT-qPCR) using TaqPath 1-Step Multiplex Master Mix (Applied Biosystems). Pre-cast TaqMan assays (Applied Biosystems) for Nuclear factor erythroid 2-related factor 2 (Nrf2), Glucose-6-phosphate dehydrogenase (G6PD), Heme Oxygenase 1 (HO-1), Interleukin-1 beta (IL-1β), Interleukin-6 (IL-6), Interferon-beta (IFN-β), and Tumor Necrosis Factor-alpha (TNF-α) were used for gene expression analysis (assay IDs: Hs00975960_m1, Hs00166169_m1, Hs01110250_m1, Hs00533490_m1, Hs01555410_m1, Hs00174131_m1, Hs01077958_s1, Hs00918082_m1, and Hs00174128_m1, respectively). Normalization was performed using GAPDH expression. Each assay was performed on the Applied Biosystems 7500 Real-Time PCR System. As regards CHAC1, and MCP-1, RT-qPCR was performed using TB Green Premix ex Taq II Master Mix (Takara Bio, France) according to the manufacturer’s instructions in a Rotor-GeneQ instrument (Qiagen, USA). The amplification conditions were for CHAC1: 95°C for 10 min, 40 cycles at 95°C for 10 s, and 60°C for 50 s; whereas for MCP-1: 95°C for 10 min, 40 cycles at 95°C for 10 s, and 64°C for 50 s, followed both by a melting curve. The expression of target genes was normalized to the reference genes: β2-Microglubulin (β2M) and GAPDH. All primers were synthesized by Sigma Aldrich (Germany): β2M, 5′-GCCTGCCGTGTGAACCAT-3′ (forward) and 5′-CATCTTCAAACCTCCATGATGCT-3′ (reverse); GAPDH, 5′-TGCCCCCATGTTTGTGATG-3′ (forward) and 5′-TGTGGTCATGAGCCCTTCC-3′ (reverse); CHAC1, 5′-TATAGTGACAGCCGTGTGGG-3′ (forward) and 5′-GCTCCCCTCGAACTTGGTAT-3′ (reverse); MCP-1 5′-AGAATCACCAGCAGCAAGTGTCC-3′ (forward) and 5′-TCCTGAACCCACTTCTGCTTGG-3′ (reverse). The fold change in specific mRNA content was calculated relative to the Mock-infected cells or to the infected untreated counterpart using the 2^−ΔΔCt^ algorithm. The results are presented as the mean fold change from at least three independent experiments ± standard deviation.

### Measurement of cytokine production

The levels of cytokines in the supernatants of Calu-3, A549-ACE2/TMPRSS2, and NHBE cells were quantified using Human Interleukin-1beta (IL-1β), Interleukin-6 (IL-6), and Tumor Necrosis Factor-Alpha (TNF-α) ELISA kits (Invitrogen, Milan, Italy) following the manufacturer’s instructions, with an overnight (o/n) incubation at 4°C instead of 2 h at room temperature (RT). A standard curve was set up for each analyte as indicated by the manufacturer and cytokine concentrations were calculated. The same samples were used for IFN-β quantification as described elsewhere (9).

### Glutathione and thiol species determination

Calu-3 cells, either mock-infected or infected with the indicated SARS-CoV-2 lineages, were treated with thiol molecules or left untreated. Samples were collected at 24 h and 48 h p.i. and thiol content was assayed. Briefly, cells were resuspended in lysis buffer (0.1% [vol/vol] Triton X-100, 0.1 M Na_2_HPO_4_, 5 mM EDTA, pH 7.5). Then, 0.1 N HCl and precipitating solution (200 mM glacial metaphosphoric acid, 6 mM Na_2_EDTA, 5 M NaCl) were added. After a 10-min incubation on ice, samples were centrifuged at 15,000 *g*. The resulting pellets were dissolved in 0.1 M NaOH, and protein concentration was determined by the Bradford assay, while the thiol species were determined in the supernatant by High-Performance Liquid Chromatography (HPLC) method, validated according to USA and European standards. The method was based on separation coupled with ultraviolet detection and pre-column derivatization with 5,5′-dithiobis-(2-nitrobenzoic acid) (DTNB) as previously described (53). Results are reported as picomoles/µmoles of total proteins.

### Protein extraction and western blot analysis

SARS-CoV-2-infected Calu-3 and A549-ACE2/TMPRSS2 cells were lysed with RIPA buffer (20 mM Tris-HCl pH7.4, 150 mM NaCl, 1% Triton X-100, 0.1% sodium dodecyl sulfate [SDS], 0.5% sodium deoxycholate [DOC]) supplemented with Complete protease inhibitor cocktail (Roche, Milan, Italy) and Halt Phosphatase Inhibitor Cocktail (Pierce, Milan, Italy). After 30 min of incubation on ice with occasionally mixing, total protein content was determined by BCA Protein Assay kit (Pierce). Total extracts (5–30 µg according to the experimental condition) were separated on 8%–10% (wt/vol) SDS-polyacrylamide gels, and proteins were transferred to the PVDF membrane and incubated with the antibodies indicated in Supplemental Materials. For consistency, the same WCLs were probed for multiple target proteins; therefore, the loading control results are identical for each analyte. HRP-linked anti-mouse or anti-rabbit (Jackson ImmunoResearch, Newmarket, UK) was used as secondary antibodies. The membranes were developed using Clarity Western ECL substrate (Bio-Rad, Hercules, CA, USA) and Bio-Rad ChemiDoc imaging system. Total proteins used for normalization were visualized on the membrane with the No-Stain Protein Labeling Reagent (Invitrogen, Italy). The quantification of total proteins and of the immunoreactive signal in each lane was performed using the Image-Lab software version 5.2.1 (Bio-Rad).

### Statistical analysis

Statistical analysis was performed by GraphPad Prism 10.0 software (GraphPad Software Inc., San Diego, California, USA) using a one-way Anova test or Welch’s *t*-test. A *P*-value < 0.05 was considered statistically significant.

## Data Availability

Data will be made available on request.

## SUPPLEMENTAL MATERIAL

Fig. S1 aac.00518-26-s0001.tif Inhibition of SARS-CoV-2 variants replication by thiol molecules.

Fig. S2 aac.00518-26-s0002.tif Thiols activity on viral protein synthesis.

Fig. S3 aac.00518-26-s0003.tif Thiol compounds counteract SARS-CoV-2 induced inflammation.

Fig. S4 aac.00518-26-s0004.tif Inflammatory pathways and CHAC1 expression by an emerging viral variant in CALU-3 and in A549-ACE2/TMPRSS2 cells.

Supplemental material aac.00518-26-s0005.docx Captions for all supplemental figures and table.

Table S1 aac.00518-26-s0006.xls Antibodies used to assess the expression of both viral and cellular proteins, along with the supplier, the relative catalogue reference and, where available, the specific clone number of the monoclonal antibody.

ASM does not own the copyrights to Supplemental Material that may be linked to, or accessed through, an article. The authors have granted ASM a non-exclusive, world-wide license to publish the Supplemental Material files. Please contact the corresponding author directly for reuse.
