# Supplementary material for: Redox-sensitive factors as targets of thiol compounds to hinder SARS-CoV-2 replication and inflammatory response
Source: Antimicrob Agents Chemother. 2026 Jul 29;70(9):e00518-26. doi: 10.1128/aac.00518-26 (PMC13712884; doi:10.1128/aac.00518-26)
Supplement: Supplementary file 1 [file aac.00518-26-s0005.docx]

**Fig S1. Inhibition of SARS-CoV-2 variants replication by thiol molecules.** Preliminary experiments were conducted to evaluate the replication kinetics of SARS-CoV-2 Wuhan-1, EG.5.1 and JN.1 strains in Calu-3 cells (A) and A549-ACE2/TMPRSS2 cells (B) at both 24h and 48h post-infection (p.i.) in cell culture supernatants from cells infected with a Multiplicity Of Infection (MOI) of 0.01. Further experiments were performed increasing the viral load for EG.5.1 and JN.1 strains up to MOI=0.05 and virus replication rate was evaluated in Calu-3 cells (C) and A549-ACE2/TMPRSS2 cells (D) at both 24h and 48h p.i. by standard titration assay. Results are presented as the mean percentage of virus release ± standard deviation (SD) from at least three independent replicates. Statistical analysis was conducted using one-way ANOVA test with Tukey correction for multiple comparison, with significance thresholds indicated as *p*<0.0001 (****), *p*<0.01 (**), and *p*<0.05 (*).

**Fig S2. Thiols activity on viral protein synthesis.** Densitometric analysis of viral Spike (S) and Nucleocapsid (N) proteins accumulation in (A) Calu-3 and (B) A549-ACE2/TMPRSS2 cells infected and thiols treated collected at 24h or 48h p.i.. Values are expressed as raw ratios of viral protein levels normalized to Actin from two (n=2) independent replicates. Protein expression levels are reported relative to untreated conditions. Statistical analysis was conducted using a one-way ANOVA test with Dunn’s multiple comparison, with significance thresholds indicated as p<0.001 (***), p<0.01 (**), and p<0.05 (*).

**Fig S3**. **Thiol compounds counteract SARS-CoV-2 induced inflammation.** Interleukin-1 beta (IL-1β), Interleukin-6 (IL-6), Tumor Necrosis Factor alpha (TNF-α) and Interferon-beta (IFN-β) mRNA levels were measured by RT-qPCR in (A and B) Calu-3 cells and (C and D) A549-ACE2/TMPRSS2 cells at 24h and 48 p.i. infected with SARS-CoV-2 or uninfected (Mock), either I-152 or I-152SdAc treated or not. The GAPDH gene was used as loading control for normalization and relative quantification via the 2^-ΔΔCt^ method. Results are expressed as mean fold change ± SD from at least three (n≥3) independent experiments. Statistical analysis was conducted using a one-way ANOVA test with Dunn’s multiple comparison, with significance thresholds indicated as *p*<0.0001 (****), *p*<0.001 (***), *p*<0.01 (**), and *p*<0.05 (*).

**Fig S4.** **Inflammatory pathways and CHAC1 expression by an emerging viral variant in CALU-3 and in A549-ACE2/TMPRSS2 cells.**

(A) whole cell lysates (WCLs) (10µg) of Calu-3 cells, either uninfected (Mock) or infected with SARS-CoV-2 Wuhan-1, EG.5.1 and JN.1 variants, were separated by SDS-PAGE. After blotting, membranes were probed with antibodies against NF-κB [P]p65/NF-κB p65. NF-kB phosphorylation level was normalized to the relative total protein expression level and values were reported in the graph on the right as the mean ± SD from three (n=3) independent experiments. (B) STAT1 and [P]STAT1 were investigated in Calu-3 WCLs (10µg) from infected or uninfected (Mock) cells, either treated or untreated with thiol compounds, by western blotting. STAT1 phosphorylation level was normalized to the relative total protein expression level and values were reported in the graph as the mean ± SD from three (n=3) independent experiments. (C) WCLs (10µg) of A549-ACE2/TMPRSS2 cells, uninfected (Mock) or infected with the EG.5.1 variant, either untreated or treated with I-152, were analyzed by western blotting using antibodies against [P]c-Fos/c-Fos, [P]JNK/JNK, and NF-κB[P]p65/NF-κB p65. Phosphorylation levels were normalized to the relative total protein expression level and values were reported in the graph as the mean ± SD from three (n=3) technical replicates. (D) MCP-1 expression was assessed in uninfected (Mock) or Wuhan-1, EG.5.1 and JN.1-infected Calu-3 cells at 48h p.i. Total RNA was extracted for RT-qPCR analysis. Relative MCP-1 mRNA levels were normalized to β2M and GAPDH and expressed as fold change relative to the corresponding untreated samples using the 2^-ΔΔCt^ method. Bar graphs represent the mean fold change ± SD from three (n=3) independent experiments. (E) CHAC1 protein levels were assessed in WCLs of A549-ACE2/TMPRSS2 cells (left panel), either mock-infected or infected with the indicated viral variants. Where indicated, thiol treatments were also performed. As further evidence of CHAC1 modulation by SARS-CoV-2, both Calu-3 and A549-ACE2/TMPRSS2 cells were infected with the recent KP.3.1 lineage or left uninfected (Mock) (right panel). After infection, complete medium or medium conditioned with I-152 or I-152SdAc was added to the cultures. Samples were collected at 48h p.i., and WCLs were prepared. CHAC1 protein levels were determined by western blotting using a specific antibody. Statistical analyses were performed using one-way ANOVA. *p*<0.001 (***/§§§), *p*<0.01 (§§), and *p*<0.05 (§). *Significance *vs* Mock; § significance *vs* corresponding untreated cells.

**Table S1. Antibodies used to detect viral and cellular proteins described within the manuscript.** A full list of the antibodies used to assess the expression of both viral and cellular proteins is provided in the table, along with the supplier, the relative catalogue reference and, where available, the specific clone number of the monoclonal antibody.
